# Survival in an era of organ preservation: an update on laryngeal cancer in Ireland

**DOI:** 10.1007/s00405-023-08055-0

**Published:** 2023-06-16

**Authors:** Gerard P. Sexton, Paul Walsh, Frank Moriarty, Paul Lennon, James Paul O’Neill

**Affiliations:** 1 Department of Otolaryngology, Head and Neck Surgery, Beaumont Hospital, Beaumont Road, Dublin 9, Ireland; 2 Royal College of Surgeons in Ireland, Dublin 2, Ireland; 3 National Cancer Registry Ireland, Cork Airport Business Park, Cork, Ireland; 4 School of Pharmacy and Biomolecular Sciences, Royal College of Surgeons in Ireland, Dublin 2, Ireland; 5 Department of Otolaryngology, Head and Neck Surgery, St James Hospital, Dublin 8, Ireland

**Keywords:** Laryngeal cancer, Head and neck cancer epidemiology, T3 laryngeal cancer, Survival, Laryngeal cancer incidence

## Abstract

**Background:**

Laryngeal cancer epidemiology has changed in recent years, with falling incidence observed internationally. Organ preservation therapies have revolutionised management, though some patients may be unsuitable and survival was noted to fall in the 2000s. This study examines trends in laryngeal cancer in Ireland.

**Methods:**

A retrospective cohort study of National Cancer Registry of Ireland data from 1994 to 2014.

**Results:**

From a cohort of 2651, glottic disease was most common (62%, *n* = 1646). Incidence rose to 3.43 cases/100,000/year for 2010–2014. 5-year disease-specific survival (DSS) was 60.6% and did not change significantly over time. Overall survival (OS) for T3 disease managed with primary radiotherapy was similar to primary surgery (HR 0.98, *p* = 0.9). DSS for T3 disease improved with primary radiotherapy (HR 0.72, *p* = 0.045).

**Conclusion:**

Incidence of laryngeal cancer in Ireland rose despite international trends, while survival changed little. Radiotherapy improves DSS for T3 disease but does not improve OS, possibly secondary to poor organ function post-radiotherapy.

## Introduction

The epidemiology of laryngeal cancer is relatively well understood, with much prior work published on the topic in both socially privileged and relatively socially deprived countries [1–3]. Despite the significant morbidity and quality-of-life impact associated with treatment of both early and advanced disease [4], survival following laryngeal cancer remains favourable relative to other varieties of head and neck cancer (HNC), such as hypopharyngeal and nasopharyngeal cancer. The incidence of laryngeal cancer has been noted in the US and Europe to be falling in recent years [5].

Laryngeal cancer of all stages was historically managed with surgery in the first instance, but landmark trials throughout the 1990s and early twenty-first century proved that organ preservation with non-operative management was a realistic possibility for many patients [6–8]. In contemporary practice, laryngeal cancer is managed using an adaptive approach taking into account specific anatomy, the extent of disease, and the overall condition and preferences of the patient [9–12]. 5-Year survival for laryngeal cancer has been consistently reported at approximately 60% for many years despite advances in treatment [10, 13]; concerningly, in some reports following the aforementioned laryngeal preservation trials, survival was noted to drop [14]. This begs the question as to whether all larynges are worth preserving.

## Aims and objectives

This study aimed to examine the epidemiology of laryngeal cancer patients in Ireland and to evaluate the response of laryngeal squamous cell carcinoma (SCC) to primary treatment modalities. Specific objectives included description of subsite data, survival trends, trends in choice of treatment modality, and the survival benefit observed in particular groups such as those with T3 disease for whom management may depend on many features.

## Materials and methods

### Study design

A retrospective cohort study was conducted using STROBE standardised reporting guidelines. The study cohort was derived from a database obtained from the National Cancer Registry of Ireland (NCRI) of HNC patients diagnosed in Ireland between January 1994 and December 2014. The length of disease-specific follow-up in this instance is until the end of 2015. This database was derived from both electronic healthcare records and physical charts which the NCRI analyses on a continual basis.

### Inclusion/exclusion criteria

The inclusion criteria were adult patients (> 18 years of age) diagnosed with a primary laryngeal cancer (as defined by the American Joint Committee on Cancer TNM Classification of Malignant Tumours (TNM) for Head and Neck Cancer 8th edition [15]) within the period specified. The primary exclusion criterion was patients with cancers occurring outside this region. Those with histological diagnoses other than SCC were excluded to ensure homogeneity of the data and reduce bias.

Despite being coded under the oropharyngeal site grouping in the International Statistical Classification of Diseases and Related Health Problems, lesions arising from the anterior epiglottis have here been included as part of the supraglottis. For analysis purposes, disease of unclear subsite has been considered together as ‘other’ and will hereafter be referred to as such.

### Statistical methods

Descriptive statistics for included participants’ baseline characteristics were generated. Kaplan–Meier survival analysis was conducted to test the survival benefit of primary radiotherapy compared with primary surgery by T stage at presentation. Primary treatment was determined by the tumour-directed therapeutic modality commenced at the earliest stage following diagnosis. TNM stage at presentation statistics and 5-year survival statistics by disease subsite and overall stage were generated. Hazard ratios adjusted for gender, age, and N- and M-staging as categorical variables were generated using multivariate Cox analysis.

The T4 cohort was also specifically analysed for the effect of primary chemoradiotherapy (defined as no more than 14 days between commencement of chemotherapy and radiotherapy without the use of primary surgical management) against primary surgery. This was performed due to the prevalent use of these modalities in combination for laryngeal preservation and as a more accurate test of the performance of non-operative management—chemotherapy would usually only be withheld in this setting due to advanced age or medical comorbidity, and thus, this is expected to mitigate some of the bias in this cohort.

Statistical analyses were conducted using Stata version 16.1 and JoinPoint version 4.9.1.0. Statistical significance was assumed at *p* < 0.05.

### Ethical considerations

Ethical approval was sought from and approved by the RCSI Research Ethics Committee. The database in question already exists, and the NCRI retains legislative authority to analyse data for research, and release data to external parties specifically for this purpose [16]. All data derived from the NCRI database are fully anonymised in line with the best practice as outlined by the Data Protection Commission, and their lawful grounds for processing same is pursuant to compliance with a legal obligation [16]. Informed consent has not been explicitly sought from any of the patients involved.

## Results

**Fig. 1:**
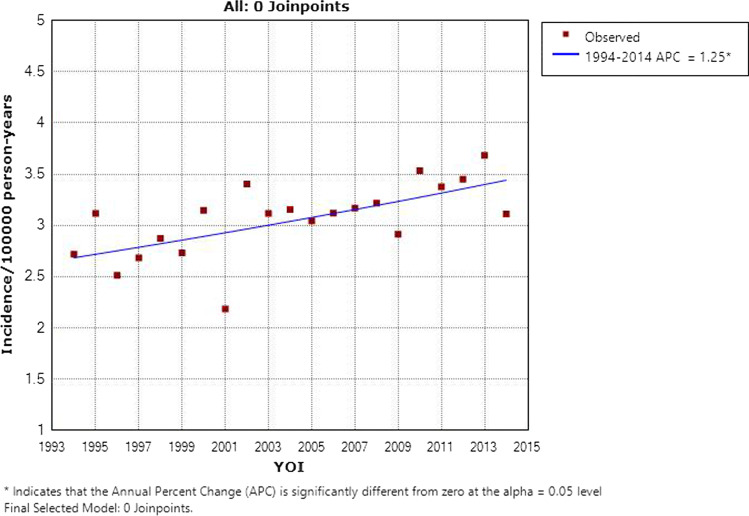
Incidence of laryngeal cancer by year of incidence (YOI) in Ireland 1994–2014

Baseline characteristics of the 2651 patients identified are summarized in Table 1. 62% (*n* = 1646) had primary glottic disease, with the supraglottis being the next most common subsite at 25% (*n* = 665). The 60–64 age group comprised the highest number of cases (*n* = 447, 16.8%) and also represented the median age group. An overwhelming majority of patients were male (85%, *n* = 2263). Figure 1 shows the incidence of laryngeal cancer rising over time—the average incidence across all years was 3.06 cases/100000/year, while the 5-year averages for 1994–1998 and 2010–2014 were 2.78 cases/100000/year and 3.43 cases/100000/year, respectively. This increase was statistically significant (*p* < 0.05) and represents a significant rise in absolute number of cases—97 cases were recorded in 1994 compared with 170 in 2013.

**Table 1:** Baseline characteristics of laryngeal squamous cell carcinoma in Ireland 1994–2014

Variable	Frequency	Percentage
Site		
Supraglottis	665	25.05
Glottis	1646	62
Subglottis	59	2.22
Other/unspecified	285	10.73
Age		
15–19	1	0.04
20–24	1	0.04
25–29	2	0.08
30–34	4	0.15
35–39	22	0.83
40–44	56	2.11
45–49	135	5.08
50–54	252	9.49
55–59	406	15.29
60–64	447	16.84
65–69	413	15.56
70–74	375	14.12
75–79	292	11
80–84	163	6.14
85 +	86	3.24
Gender		
Female	388	14.6
Male	2263	85.4

TNM stage at presentation statistics overall and by subsite are summarized in Table 2. T1 and T2 diseases were the most commonly encountered overall (31% and 24.4%, respectively) and particularly in the glottis (42.4% and 21.9%, respectively). The glottis also represented the most common source of T3 (*n* = 199, 47.6%) and T4 (*n* = 117, 33.9%) disease despite higher prevalence of such disease within other subsites. N0 disease was recorded in 55.2% (*n* = 1582). This was higher in the glottis (65%, *n* = 1068) than the supraglottis (44.5%, *n* = 296) and subglottis (54.2%, *n* = 32). The prevalence of all N stages of disease was lower in glottic disease than the corresponding level in either supraglottic or subglottic disease. M1 disease was present in 3.3% (*n* = 87).

**Table 2:** Stage statistics at presentation of laryngeal cancer in Ireland 1994–2014

Stage	Supraglottic	Glottic	Subglottic	Other	Total
T stage					
T1	74 (11.1%)	696 (42.4%)	7 (11.9%)	45 (15.9%)	822 (31.0%)
T2	222 (33.4%)	359 (21.9%)	20 (33.9%)	47 (16.6%)	648 (24.4%)
T3	157 (23.6%)	199 (12.1%)	9 (15.3%)	53 (18.7%)	418 (15.8%)
T4	131 (19.7%)	117 (7.1%)	14 (23.7%)	83 (29.2%)	345 (13.0%)
Tx	81 (12.2%)	272 (16.6%)	9 (15.3%)	56 (19.7%)	418 (15.8%)
N stage					
N0	296 (44.5%)	1068 (65.0%)	32 (54.2%)	122 (43.0%)	1518 (57.3%)
N1	102 (15.3%)	66 (4.0%)	9 (15.3%)	32 (11.3%)	209 (7.9%)
N2	155 (23.3%)	74 (4.5%)	7 (11.9%)	50 (17.6%)	286 (10.8%)
N3	13 (2.0%)	3 (0.2%)	0 (0.0%)	3 (1.1%)	19 (0.7%)
NX	99 (14.9%)	432 (26.3%)	11 (18.6%)	77 (27.1%)	619 (23.4%)
M stage					
M0	352 (52.9%)	859 (52.3%)	30 (50.9%)	133 (46.8%)	1374 (51.8%)
M1	41 (6.2%)	22 (1.3%)	1 (1.7%)	23 (8.1%)	87 (3.3%)
MX	272 (40.9%)	762 (46.4%)	28 (47.5%)	128 (45.1%)	1190 (44.9%)
Total	665 (100%)	1643 (100%)	59 (100%)	284 (100%)	2651 (100%)

### Survival

5-year disease-specific survival (DSS) statistics by overall stage and subsite are presented in Table 3. 5-year DSS from laryngeal cancer was 60.6% for the period studied, though the glottic region was significantly higher at 71.8% compared with the subglottic region at 40%. 85.9% of those with stage I disease survived to 5 years compared with 5.8% of those with stage IVC. There was no notable difference in 5-year DSS between male and female patients (60.2% vs 62.4%, *χ*^2^ = 0.41, *p* = 0.52).

**Table 3:** 5-Year disease-specific survival for laryngeal cancer in Ireland 1994–2014 by stage

Disease stage	Supraglottic (%)	Glottic (%)	Subglottic (%)	Other (%)	Total (%)
Stage I	86.8	87.0	50.0	70.8	85.9
Stage II	56.6	71.5	72.7	54.8	66.1
Stage III	42.7	46.1	30.0	53.3	45.2
Stage IV	24.8	34.8	22.2	25.9	28.2
Stage IVC	4.4	0.0	0.0	12.5	5.8
Overall	42.6	71.8	40.0	40.6	60.6

**Fig. 2:**
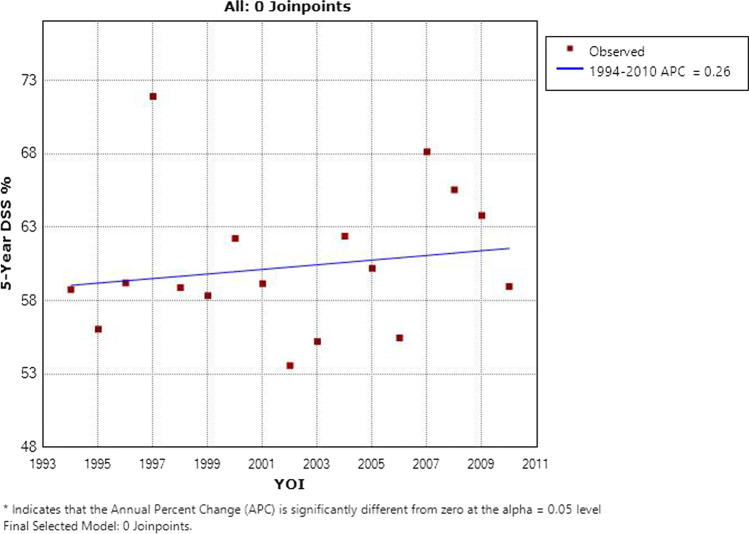
5-Year disease-specific survival in laryngeal cancer by year of incidence in Ireland 1994–2014

**Fig. 3:**
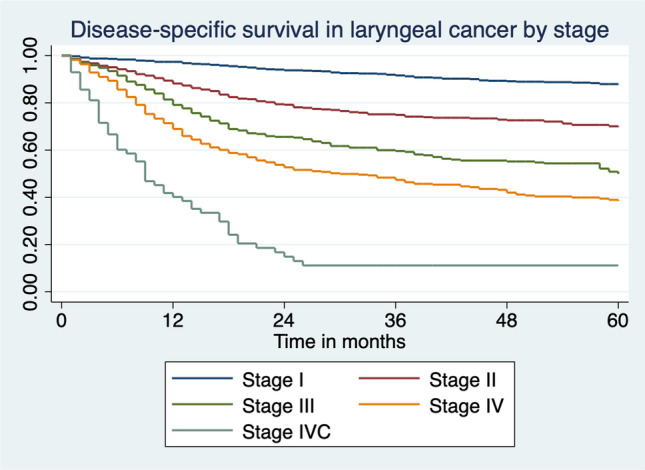
Kaplan–Meier graph of disease-specific survival by stage in laryngeal cancer in Ireland 1994–2014

Survival was noted to increase marginally over time, as shown in Fig. 2, though statistical significance was not achieved (*p* = 0.48). When broken down by stage, this increase was most notable for stage I disease (*p* = 0.007) and was negligible for the remainder. A Kaplan–Meier curve of DSS by stage is shown in Fig. 3.

### Primary radiotherapy versus surgery

**Fig. 4:**
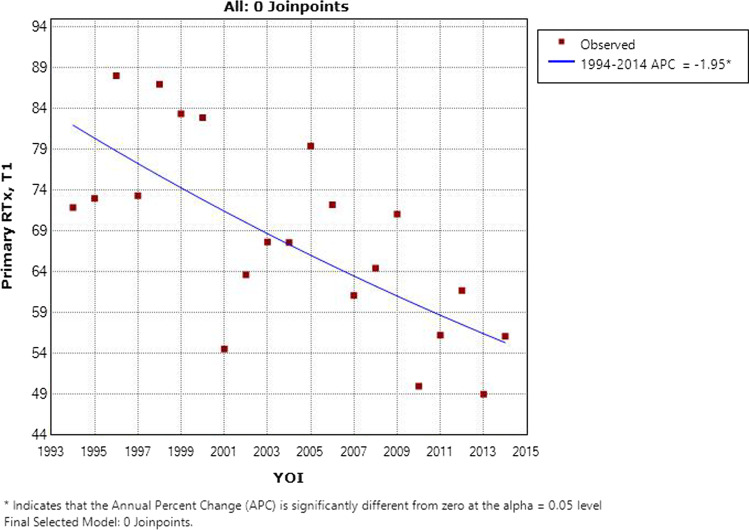
Percentage of T1 laryngeal cancer managed with primary radiotherapy by year of incidence in Ireland 1994–2014

**Fig. 5:**
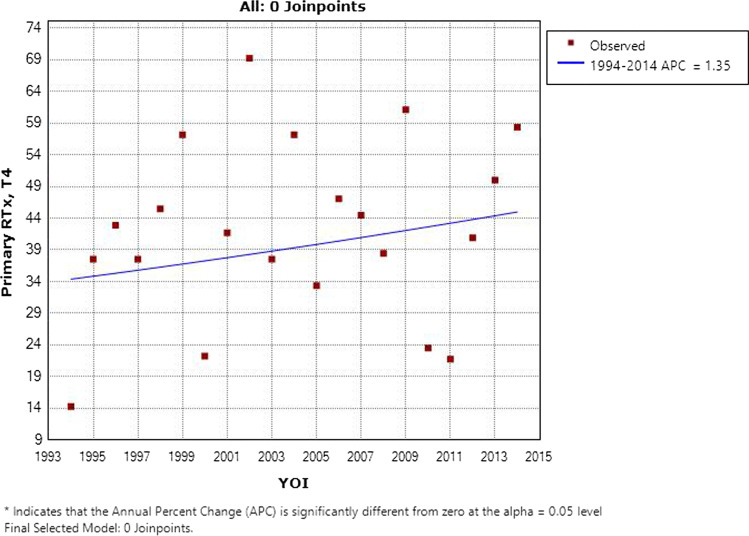
Percentage of T4 laryngeal cancer managed with primary radiotherapy by year of incidence in Ireland 1994–2014

The results of Cox multivariate regression for primary radiotherapy versus primary surgery by subsite and T stage are shown in Table 4. 67% (*n* = 505) of T1 disease was managed with radiotherapy; a similar proportion of T2 (74%, *n* = 460) and T3 (78%, *n* = 303) disease was managed as such. T4 disease was managed with primary surgery in 58% (*n* = 168), with the remainder receiving primary radiotherapy. The proportion of T1 and T4 disease managed with primary radiotherapy by year of incidence is shown in Figs. 4 and 5. Over time, there was a statistically significant increase in the use of primary surgery rather than radiotherapy for T1 disease (*p* = 0.003). Use of primary radiotherapy for T4 disease rose significantly in the late 1990s, but later in the study period, this approach became less prevalent. No trends of note were identified for T2 or T3 disease.

**Table 4:** Primary radiotherapy versus surgery for laryngeal cancer in Ireland 1994–2014 by site and stage

T stage	Modality		Overall survival		Disease-specific survival	
	RTx	Surgery	Hazard ratio	*P* value	Hazard ratio	*P* value
All sites						
T1	505 (67%)	251 (33%)	1.25 (0.97–1.61)	0.089	1.23 (0.83–1.83)	0.297
T2	460 (74%)	160 (26%)	0.77 (0.61–0.96)	0.018	0.78 (0.59–1.04)	0.086
T3	303 (78%)	85 (22%)	0.98 (0.74–1.30)	0.900	0.72 (0.52–0.99)	0.045
T4	123 (42%)	168 (58%)	1.18 (0.90–1.56)	0.234	1.16 (0.84–1.61)	0.365
Supraglottis						
T1	46 (67%)	23 (33%)	3.87 (1.56–9.61)	0.004	4.67 (1.28–17.04)	0.020
T2	142 (68%)	67 (32%)	0.75 (0.53–1.05)	0.091	0.88 (0.57–1.36)	0.563
T3	117 (81%)	28 (19%)	0.81 (0.50–1.31)	0.386	0.61 (0.35–1.07)	0.085
T4	63 (60%)	42 (40%)	0.96 (0.61–1.51)	0.855	0.85 (0.50–1.46)	0.562
Glottis						
T1	427 (67%)	209 (33%)	1.25 (0.93–1.67)	0.139	1.31 (0.81–2.13)	0.275
T2	279 (81%)	67 (19%)	0.84 (0.59–1.19)	0.321	0.72 (0.46–1.13)	0.155
T3	145 (78%)	40 (22%)	1.30 (0.83–2.04)	0.253	0.88 (0.53–1.47)	0.636
T4	38 (38%)	61 (62%)	1.29 (0.76–2.20)	0.347	1.45 (0.77–2.74)	0.250
Subglottis						
T1	5 (83%)	1 (17%)	–	–	–	–
T2	13 (68%)	6 (32%)	1.00 (0.10–10.04)	0.999	0.76 (0.05–10.89)	0.843
T3	5 (56%)	4 (44%)	11.29 (0.6–211.8)	0.105	–	–
T4	5 (38%)	8 (62%)	13.27 (1.1–155.2)	0.039	8.94 (0.67–120.1)	0.098
Other						
T1	27 (60%)	18 (40%)	0.55 (0.24–1.24)	0.149	0.39 (0.13–1.18)	0.096
T2	26 (57%)	20 (43%)	1.03 (0.48–2.22)	0.945	1.39 (0.54–3.60)	0.498
T3	36 (73%)	13 (27%)	0.45 (0.21–0.96)	0.039	0.29 (0.12–0.67)	0.004
T4	17 (23%)	57 (77%)	0.87 (0.45–1.67)	0.681	0.88 (0.39–2.00)	0.762

T1 disease showed no notable difference in both overall survival (OS) (HR 1.25, *p* = 0.089) and DSS (HR 1.23, *p* = 0.297) associated with the use of radiotherapy over surgery. Supraglottic disease showed statistically significantly worse DSS (HR 4.67, *p* = 0.02) with the use of radiotherapy.

T2 disease showed statistically significantly improved OS with the use of radiotherapy (HR 0.77, *p* = 0.018). Statistical significance was not achieved for DSS (HR 0.78, *p* = 0.086). This effect was strongest in the supraglottic group (HR 0.75, *p* = 0.091). Exclusion of node positive disease yielded a cohort of 288 with T2N0 disease; there was no difference in OS (HR 0.93, *p* = 0.61) or DSS (HR 0.97, *p* = 0.86) between the treatment groups for this subgroup.

T3 disease showed no statistically significant difference in OS between radiotherapy and surgery groups (HR 0.98, *p* = 0.9). There was a statistically significant benefit to DSS associated with the use of radiotherapy in the same group (HR 0.72, *p* = 0.045). The ‘other’ site group was the only subsite that achieved statistical significance for DSS (HR 0.29, *p* = 0.004), though this was narrowly missed in the supraglottis (HR 0.61, *p* = 0.085).

T4 disease showed no notable difference in either OS (HR 1.18, *p* = 0.234) or DSS (HR 1.23, *p* = 0.297) associated with the use of radiotherapy over surgery. No statistically significant difference was noted among any of the subsites with the exception of the subglottis, where radiotherapy was noted to decrease OS (HR 12.37, *p* = 0.039). Primary chemoradiotherapy did not produce a statistically significant change in OS (HR 0.85, *p* = 0.51) or DSS (HR 0.74, *p* = 0.34) when compared with primary surgery.

## Discussion

The epidemiology of laryngeal cancer has developed considerably in the recent past, with low- to middle-income countries such as India and Pakistan noting increasing incidence rates and more advanced disease stages at presentation [1]. By comparison, in relatively socioeconomically privileged countries, the incidence has either gone largely unchanged or has declined [2, 17]. This pattern is not replicated in the presented data, with incidence in Ireland continuing to rise at least during the observed time period. The reasons for this are unclear—multiple high profile public health measures, including a national ban on smoking in the workplace and enclosed public spaces, have been instituted in Ireland during this time frame, though clearly there will be a lag time in the effects of these measures [18]. The incidence internationally was recently estimated at 2.76 cases/year per 100,000 inhabitants [5]; the Irish incidence was notably higher than this at almost every time point. This phenomenon also occurred during a time of significant increase in the population of Ireland from 3.56 million in 1994 to 4.62 million in 2014 [19]—clearly, this represents a significant increase in workload. Notably, the universally observed preponderance of male gender among laryngeal cancer patients is in contrast with some recent reports of poorer survival relative to their female counterparts [3]—this finding was not observed here, indicating that with adequate and equitable access to healthcare, there is not necessarily a gender bias in laryngeal cancer survivorship.

Conservation of the anatomical larynx, as famously described in the landmark publication by Wolf et al. [6], now forms the basis for much of the treatment rationale for advanced laryngeal cancer. It additionally represents one of the more controversial areas in head and neck cancer; despite a strong body of evidence that T4 disease mandates primary ablative surgery [7, 20–22], there are multiple recent reports of the successful use of concurrent chemoradiotherapy regimes for organ preservation in such disease [23–25]. No notable difference in survival was detected in the reported data for patients with T4 disease treated with primary surgery or radiotherapy. It is not clear why this was the case—differentiation of subtyping into T4a and T4b was not available which would have been useful for distinguishing those with probable unresectable primary disease and correction for additional variables as described did not further clarify the issue. In addition, specifically comparing primary chemoradiotherapy against primary surgery did not produce a significant difference. Important changes in the TNM staging system in 2003 likely introduce some bias, as minor erosion of the thyroid cartilage was redesignated as T3 rather than T4 disease, in essence rendering T4 disease more homogeneously advanced [26]. This has previously been noted to have ramifications for registry-based data [27]. Primary surgery currently represents the standard of care in Ireland for resectable T4a laryngeal cancer without metastatic disease—further inquiry is warranted to explain the lack of a significant difference in survival. 42% of cT4 patients underwent non-surgical management during the period analysed. This follows the international trend towards non-surgical management of such patients during the same time period where survival in the United States was noted to fall [14]. It can only be concluded that multiple factors were at play in the survival in Ireland remaining stable overall.

For early stage laryngeal cancer, specifically T2 or earlier, choice of therapy is largely dictated by the specific anatomy of the disease [28]. In the presented data, survival for T1 lesions was shown to be similar regardless of choice of therapy, while the same was true of T2N0 lesions. This reflects the role that patient choice, available expertise, and disease anatomy often play in such decisions. Supraglottic lesions had worse DSS where radiotherapy was employed—this may reflect selection bias, as most supraglottic lesions amenable to laser resection would be smaller and more homogeneous compared to the wider array of T1 glottic lesions. For T2 disease, overall radiotherapy was associated with significantly improved survival, especially in the supraglottic subgroup. A potential lurking variable here is that failure of local control in either modality results in disparate outcomes—where a positive margin following laser excision could be expected to respond to adjuvant radiotherapy, incomplete response to primary radiotherapy will mandate salvage surgery which is itself a poor prognostic marker. It is interesting to note the increasing prevalence of primary surgery in T1 laryngeal cancer as this modality became more available in Ireland. This occurred without any notable decline in survival for T1 or T2 disease.

For T3 disease, primary radiotherapy with or without chemotherapy represents the standard of care in Ireland and in other countries in Europe [29, 30]. It is known that some with T3 disease undergo surgery for reasons including for salvage or in the setting of a non-functional larynx [29–31]. DSS was significantly improved for T3 disease by the use of primary radiotherapy as opposed to primary surgery; this would be the expected finding as those requiring primary surgery would be expected to have more advanced disease. However OS was the same in both groups, suggesting that patients receiving primary radiotherapy are dying at similar rates for reasons other than disease progression. A possible explanation for this is poorly functioning, post-radiotherapy larynges which are at increased risk of aspiration of secretions and oral intake due to impaired airway protection. This being the case would be highly relevant as it would further emphasise the findings of Kim et al. in 2018 that OS in T3 disease was no different between the total laryngectomy group and the laryngeal preservation group [32]. The purpose of this observation is not to suggest that primary radiotherapy should not represent a standard of care in T3 disease, but rather that further investigation is warranted to systematically identify those with T3 disease who are unlikely to benefit from laryngeal preservation to the extent that primary surgery should instead be considered.

## Conclusion

Despite innovations in laryngeal surgery and radiotherapy and concerning international survival trends, DSS following laryngeal cancer in Ireland remains largely unchanged at 60.6%. The incidence has increased to approximately 3.43 cases/100000/year. Primary radiotherapy improves DSS for T3 disease relative to primary surgery but does not improve OS—this may be due to poor organ function post-radiotherapy. No survival difference was noted between primary surgery and chemoradiotherapy for T4 disease.

## Data Availability

Stored and maintained by National Cancer Registry Ireland.

## Code availability

Stata 16.1.
